# Transitory Shifts in Skin Microbiota Composition and Reductions in Bacterial Load and Psoriasin following Ethanol Perturbation

**DOI:** 10.1128/msphere.00171-22

**Published:** 2022-06-21

**Authors:** Charis R. Saville, Aline Metris, Gavin J. Humphreys, Catherine O’Neill, Paul Barrett, Judith Fernandez-Piquer, Andrew J. McBain

**Affiliations:** a Division of Pharmacy and Optometry, School of Health Sciences, Faculty of Biology, Medicine and Health, The University of Manchester, United Kingdom; b Unilever, Safety & Environmental Assurance Centre (SEAC), Sharnbrook, United Kingdom; c Division of Musculoskeletal & Dermatological Sciences, School of Biological Sciences, Faculty of Biology, Medicine and Health, The University of Manchester, United Kingdom; University of Nebraska Medical Center

**Keywords:** microbiome, recolonization stability, 16S sequencing, clinical study, risk assessment

## Abstract

Personal care and hygiene regimens may substantially alter the composition of the skin microbiota through direct and indirect mechanisms. An understanding of the timescales of commensal skin microbiota reestablishment following perturbation is required to inform consumer safety risk assessment, and support product development. In the current investigation, the microbiota of the volar and dorsal forearm of 10 volunteers was sampled immediately before and after wiping with 70% ethanol and at up to 24 h afterwards. Quantitative PCR and amplicon sequencing were used to measure microbial load and composition, and concentrations of the antimicrobial peptide psoriasin were measured using an enzyme-linked immunosorbent assay (ELISA). Ethanol wiping significantly reduced the total bacterial abundance at 2 h post-wipe. Recovery was observed after 6 h for total bacterial populations and for Staphylococcus epidermidis depending on the site tested. Microbiome diversity recovered by 6 h after wiping. Psoriasin concentrations were highly variable between volunteers, ranging from 42 to 1,569 ng/mL, and dorsal concentrations were significantly higher than volar concentrations (*P* < 0.05). For most of the volunteers, the application of ethanol decreased psoriasin concentrations, particularly for the dorsal samples, but the overall effect was not significant. This work extends observations of skin microbiome stability and demonstrates resilience in a key antimicrobial peptide.

**IMPORTANCE** An understanding of the timescales of commensal skin microbiota reestablishment following perturbation is required to inform consumer safety risk assessment and support product development. Following ethanol exposure, total bacterial populations and microbiome diversity recovered after 6 h. For most of the volunteers, the application of ethanol decreased psoriasin concentrations, but the overall effect was not significant. This work extends observations of skin microbiome stability and demonstrates resilience in a key antimicrobial peptide.

## INTRODUCTION

The microbiota of the skin can affect host health both positively and negatively (1, 2). Positive effects include the suppression of adventitious pathogens, which is probably mediated through factors which include the antimicrobial peptides of both microbial (3) and host (4, 5) origin. Antimicrobial peptides are commonly produced by skin commensal bacteria, including staphylococci (6–9). Additionally, bacterial fermentation products of bacterial skin commensals, including fatty acids, have been reported to control the growth of opportunistic pathogens such as Cutibacterium acnes (10). Other protective effects may be mediated through the skin barrier, where, for example, Staphylococcus epidermidis may upregulate tight junction expression in keratinocytes (11); this mechanism is apparently mediated by lipoteichoic acids and triggered through Toll-like receptors (TLR) (12). Similar beneficial effects on the tight junction-associated barrier have been reported to be stimulated by the TLR2 ligand peptidoglycan and mediated through TLR2 activation (13).

A role for the microbiota of the skin in shaping epidermal immune function has been proposed where protective immunity to the cutaneous pathogen Leishmania major has been observed to depend on the skin microbiota and interleukin-1 signaling in mice (14). Meisel et al. (15) profiled skin transcriptomes of normally colonized and gnotobiotic mice, reporting that over 2,800 genes were regulated differentially during colonization, particularly those associated with immune response, epidermal differentiation, and cytokine activity, including Toll-like receptors, antimicrobial peptides, and immune functions. Taken together, studies including those summarized above indicate that commensal microorganisms of the skin are involved in multiple functions relevant to the epithelial barrier, homeostasis, and protection from infection.

Despite the increasing understanding of interactions between skin microbiota and human host, the stability of the skin microbiota under perturbation is incompletely understood. The microbiota of the skin is exposed to a variety of perturbing factors, including UV light (16), friction, and hygiene regimes (17) that may include the use of antimicrobial compounds (18), and antisepsis (19, 20). Such factors can potentially influence the epidermal microbiota directly through antimicrobial effects (18) or indirectly by altering the epidermal environment, and potentially by altering the concentrations of antimicrobial peptides.

An understanding of the timescales of skin microbiota reestablishment following perturbation is required to inform consumer safety risk assessment and support the development of microbiome-friendly formulations for hygiene (21). A better understanding of the types of microbiota and bacterial load shifts can help build data for microbiome-based risk assessment in personal care. The study of indirect effects, such as the disruption of antimicrobial peptides on the skin such as psoriasin, which has been shown to be functionally important (22), could also be used as an indicator for such assessments. Psoriasin was originally named due to its high levels of expression in the keratinocytes of psoriasis patients (23, 24). However, it has since been discovered to have antimicrobial activity against several microorganisms, including Escherichia coli, Pseudomonas aeruginosa, and Staphylococcus aureus (23). It is highly abundant on the skin and thus acts as a natural defense, probably protecting the skin from colonization by pathogenic bacteria. It is measurable by enyzme-linked immunosorbent assay (ELISA) (25) and therefore offers an ideal candidate for studying the effects of hygiene on antimicrobial peptides, which could have direct implications for the reestablishment of commensal microorganisms. In this investigation we profiled the epidermal microbiota longitudinally and measured concentrations of a key epidermal antimicrobial peptide on the volar and dorsal forearms of 10 individuals immediately before and after wiping with 70% ethanol and at different time intervals for up to 24 h.

## RESULTS

### Stability in the baseline forearm microbiome.

**FIG 1:**
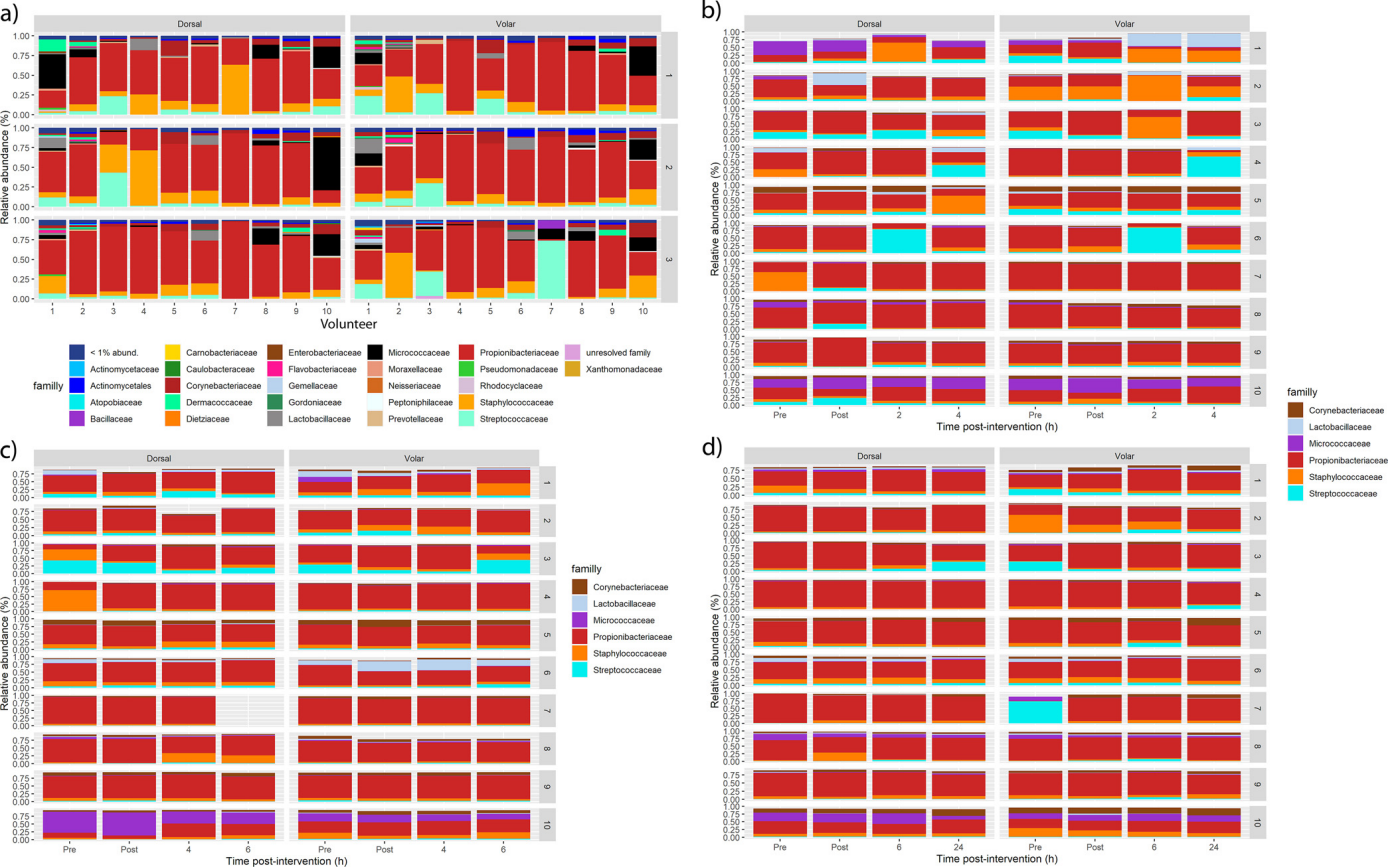
Relative bacterial abundance at the family level (a) at the baseline per panelist (horizontal axis) at dorsal and volar forearm sites, with each row representing a visit (visits 1, 2 and 3). Families with an abundance of less than 1% are not displayed. Propionibacteriaceae are dominant for most individuals at most visits. (b) Short-term effects of ethanol wiping on the 6 most abundant families at visit 1, and (c and d) longer-term effects on the 6 most abundant families at visits 2 and 3, respectively. No systematic trend could be identified.

**FIG 2:**
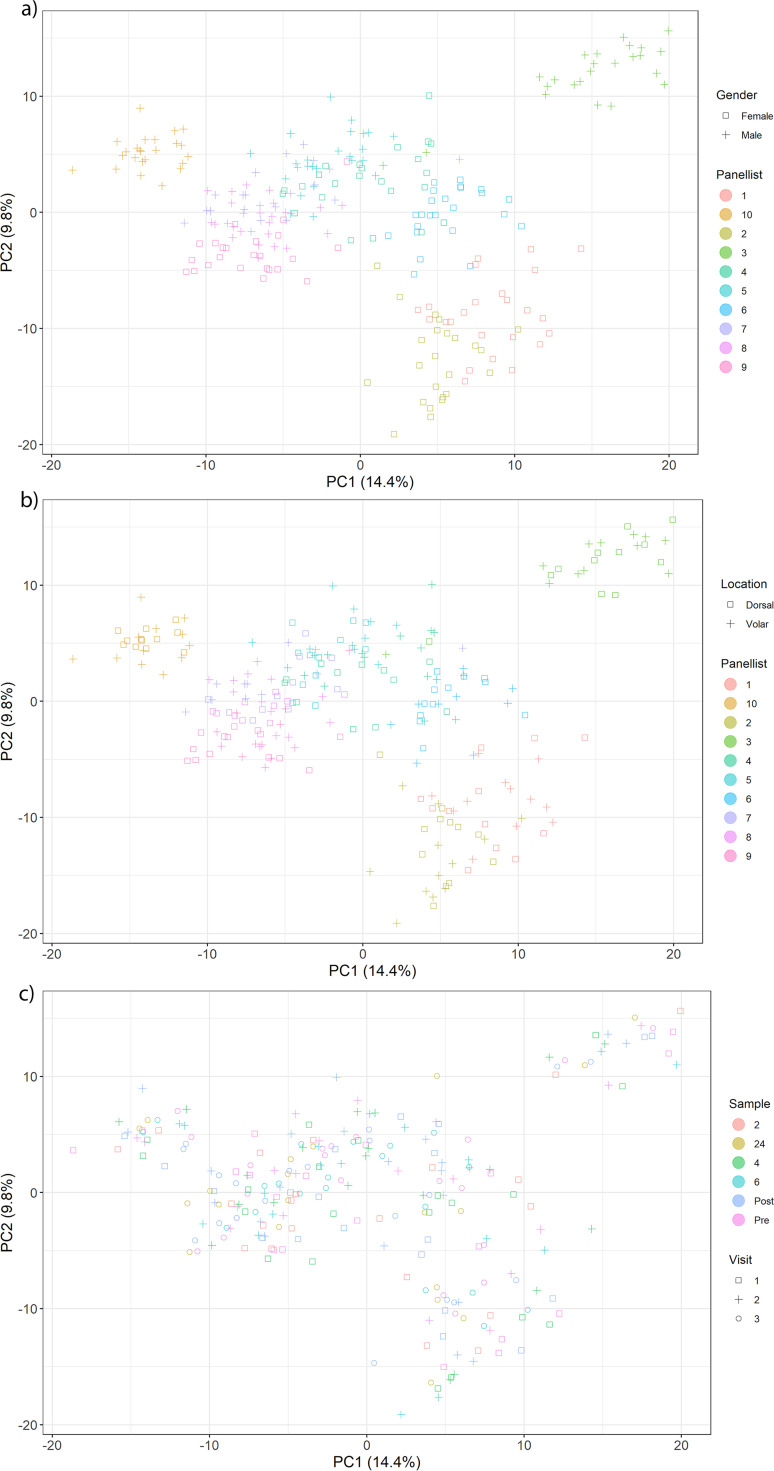
Principal-component analysis of the Aitchison distance between microbiomes, shown by (a and b) individual panelists and (c) sampling times. Microbiomes are grouped by (a) gender and (a and b) individual, but not by (b) sampling location or (c) sample.

Baseline samples taken for each individual at the three sampling visits were compared to assess the interpersonal stability of the skin microbiome. A comparison of the individual microbial profiles indicates stability across time (Fig. 1a). The baseline microbiomes of each individual were highly stable, and volar and dorsal sample profiles were highly similar within individuals. *Cutibacterium* (formerly *Propionibacterium*), *Corynebacterium*, Streptococcus, and Staphylococcus dominated most samples. A comparison of the visit-to-visit and inter-individual variability revealed a higher degree of variability between individuals than between visits, as shown in the principal-component analysis (PCA) (Fig. 2).

### Bacterial profiles and resistance to ethanol wiping.

Microbial community profiles remained relatively stable despite topical ethanol application, with a clear dominance of *Cutibacterium* (Propionibacteriaceae family, Fig. 1b to d). However, early differences were apparent in some individuals. Individuals 3 and 6 appeared to have an increased abundance of Streptococcus at 2 h post-wiping, which is seen in individual 4 at 4 h (Fig. 1b). After wiping, individuals 2 and 3 had an initial increase in the relative abundance of Staphylococcus, whereas individual 1 had a large increase in *Lactobacillus* (Fig. 1b). Only two operational taxonomic units (OTUs) were significantly affected directly after treatment and up to 2 h, as indicated by a hierarchical model (26); Kocuria rhizophila is a soil microorganism representing less than 1% of the total OTUs and Rothia dentocariosa an oral and respiratory tract commensal representing less than 0.1% of the total OTUs, so they are probably external contaminants and unlikely to be a part of a systematic skin microbiome shift. By the later time points (6- and 24-h), captured in the second and third visits, no such significant increases in specific genera were observed (Fig.1c and d).

**FIG 3:**
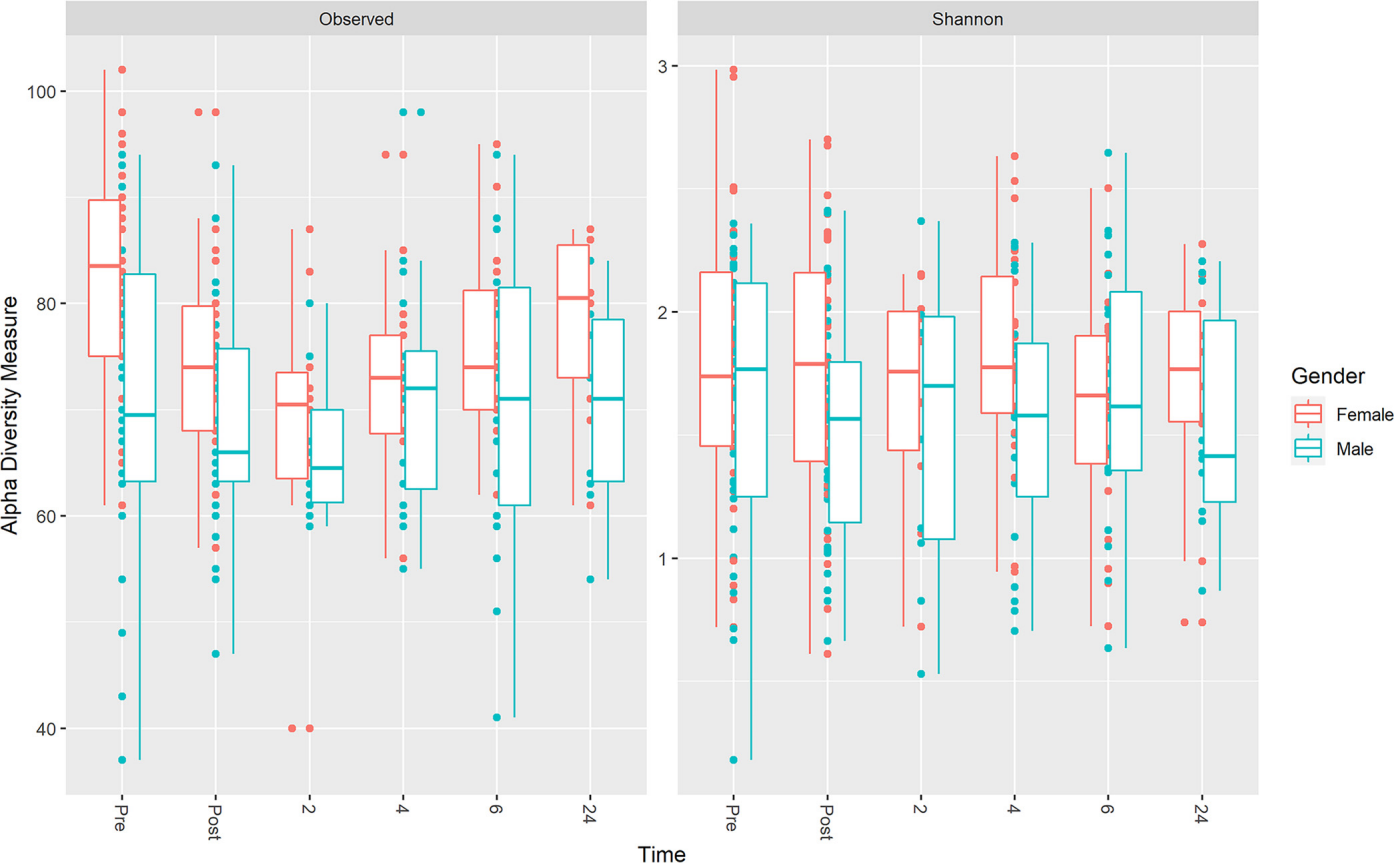
Variation of alpha diversity during treatment and recovery. Diversity is systematically higher for females. Observed diversity (the number of operational taxonomic units) decreases with treatment and recovers in approximately 6 h; this trend is significant in females. The trend is less obvious for the Shannon diversity. Significance levels are given in Table S2 in the supplemental material.

Richness per sample, as measured by the number of operational taxonomic units, was systematically higher for females than males (Observed alpha diversity in Fig. 3). Richness decreased with treatment for both males and females and recovered in 6 h, although this trend was only significant for females (Table S2 in the supplemental material). A similar but less significant trend was observed for Shannon diversity, which accounts for phylogenetic relationships between OTUs as well as richness.

10.1128/msphere.00171-22.2 TABLE S2 Statistical analysis of alpha-diversity showing that, for females, observed alpha diversity decreases significantly (FDR at cutoff limit of 10%) with ethanol wiping and was recovered in 6 h. The same trend was observed in males (Fig. 2), but this was not significant. The Shannon diversity exhibits a similar trend. The location has also a significant effect. FDR values were obtained with MaasLin2 hierarchical models. Download Table S2, DOCX file, 0.01 MB. Copyright © 2022 Saville et al. 2022 Saville et al. https://creativecommons.org/licenses/by/4.0/ This content is distributed under the terms of the Creative Commons Attribution 4.0 International license.

Principal-component analysis of the Aitchison distance between microbiomes (Fig. 2a) revealed that the individual was the most significant discriminatory effect (analysis of similarities [ANOSIM] *R* = 0.85 at a significance level of 0.01), with males and females clustering together (ANOSIM *R* = 0.21 with a significance of 0.01). The site tested (dorsal or volar) was not significant overall but tended to group per individual (Fig. 2b). The visit and sample time had no discriminatory effects compared to the other factors (Fig. 2c).

Overall, the data suggest that the microbiome load and richness recovered between 6 and 24 h after ethanol wiping, and the bacterial relative abundance, were not systematically affected by treatment.

### Recovery of bacterial abundance following exposure of the skin with 70% ethanol.

**FIG 4:**
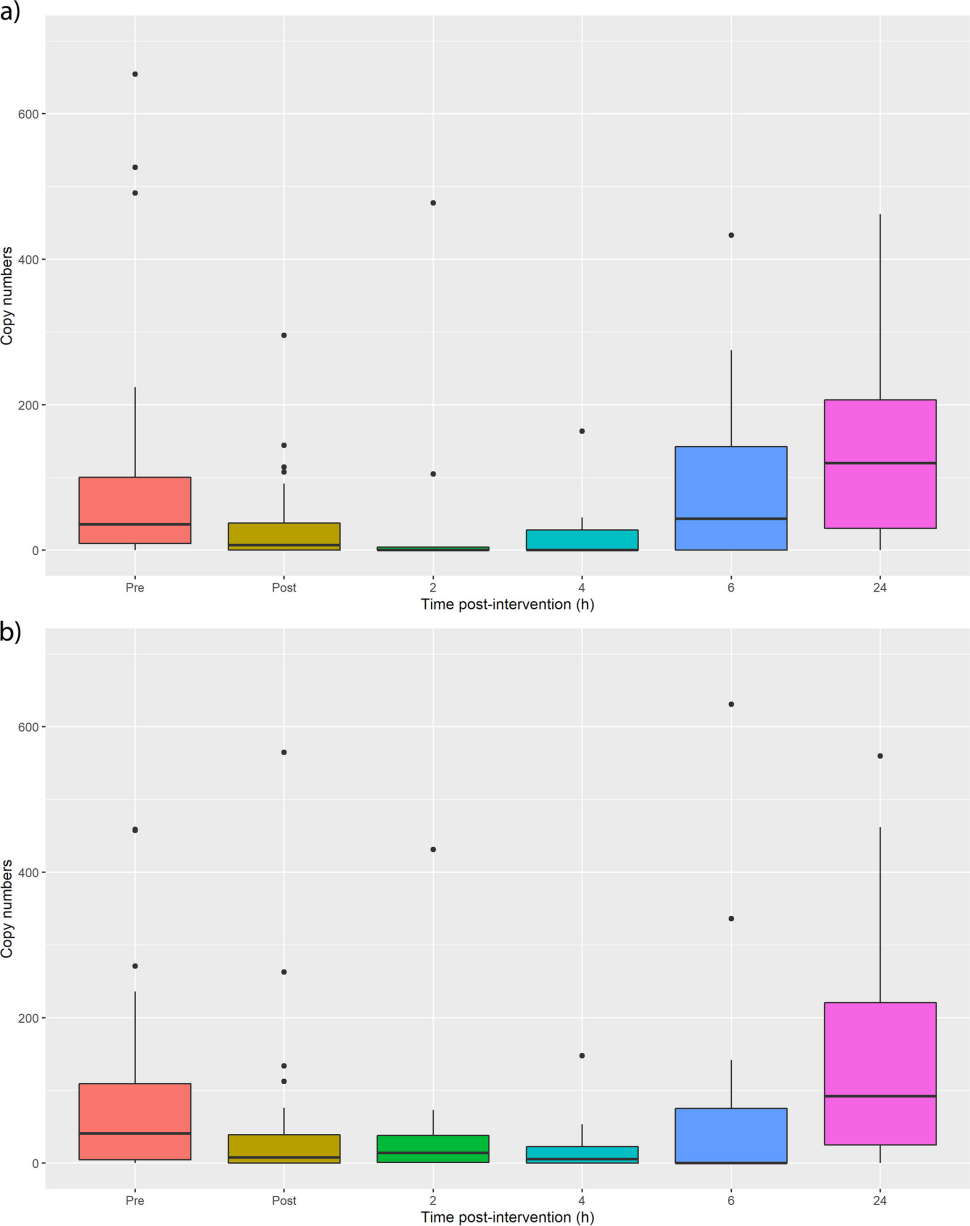
Total bacterial abundance before and after wiping the (a) volar forearm and (b) dorsal forearm, as measured by quantitative PCR. Ethanol wiping decreases the bacterial load, which takes 6 h to recover, although this is only statistically significant for the volar forearm. Statistics are provided in Table S1.

**FIG 5:**
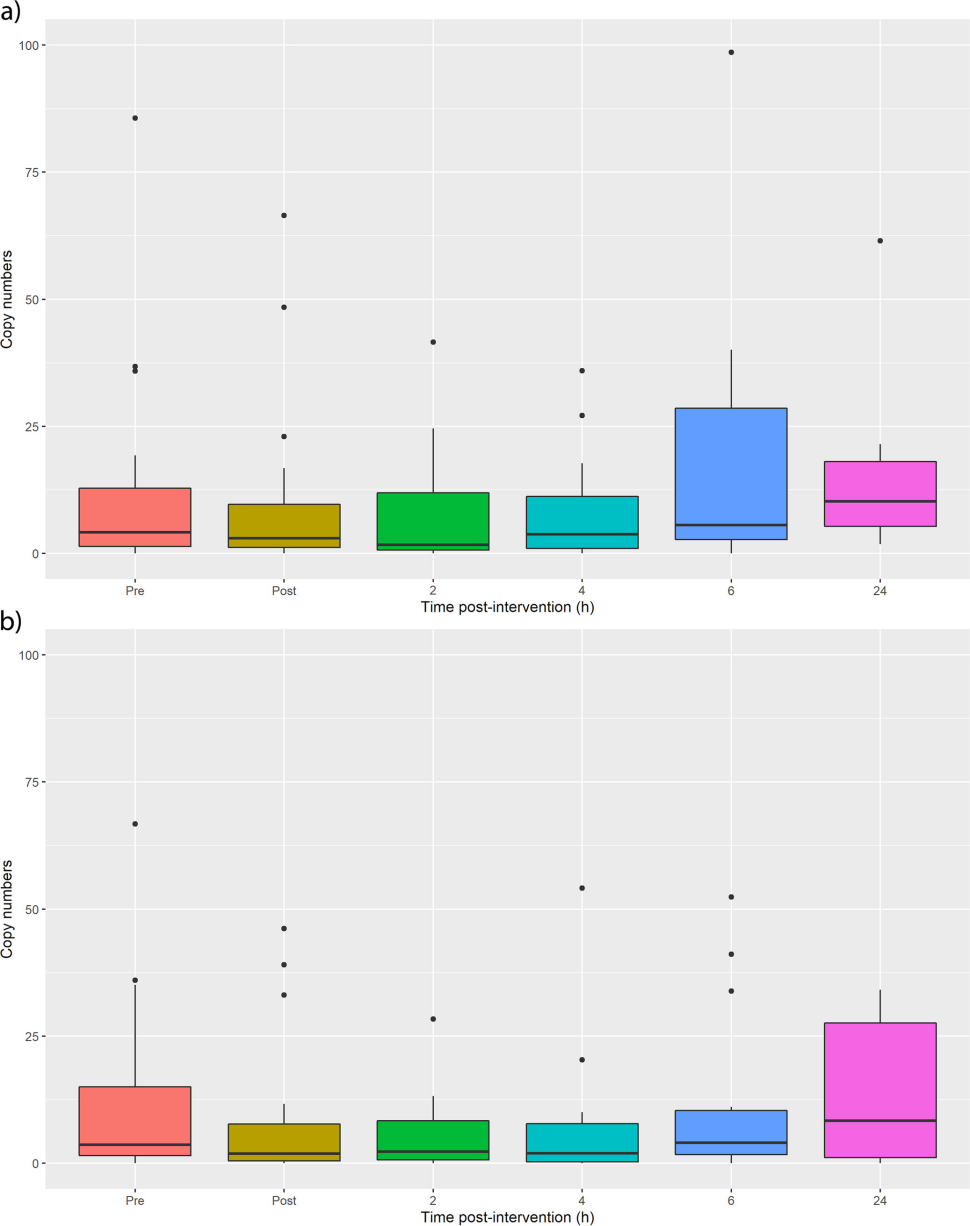
Staphylococcus epidermidis abundance before and after wiping the (a) dorsal and (b) volar forearm. A significant decrease is observed upon ethanol wiping but recovery is unclear because of the low copy numbers. The figure suggests that they recover in approximately 6 h, similarly to total bacterial abundance. Statistics are provided in Table S1.

Quantitative PCR data showed a reduction in total eubacterial 16S following ethanol wipe (*P* = 0.04 for volar [Fig. 4a] and 0.01 for dorsal [Fig. 4b], Wilcoxon paired test) and recovered in 6 h (*P* = 0.29 and 0.35 for volar and dorsal, respectively). Quantitative PCR targeting Staphylococcus epidermidis showed a significant reduction (*P* = 0.003 for dorsal [Fig. 5a] and *P* = 0.03 for volar [Fig. 5b]), but recovery was less obvious, with data indicating minimum abundance at 2 to 4 h and maximum after 6 to 24 h depending on the sampling site.

10.1128/msphere.00171-22.1 TABLE S1 Statistical analysis of qPCR data showing that numbers of both total eubacteria and *S. epidermidis* tend to drop with treatment but only significantly for total bacteria and volar forearm. This decrease recovered in 6 h. FDR values obtained with MaasLin2 hierarchical models. The time points (pre, post, 2h, 4h, 6h, 24h) were considered as fixed effects while the visit and the individual were considered as random effects. Download Table S1, DOCX file, 0.02 MB. Copyright © 2022 Saville et al. 2022 Saville et al. https://creativecommons.org/licenses/by/4.0/ This content is distributed under the terms of the Creative Commons Attribution 4.0 International license.

### Psoriasin concentrations.

**FIG 6:**
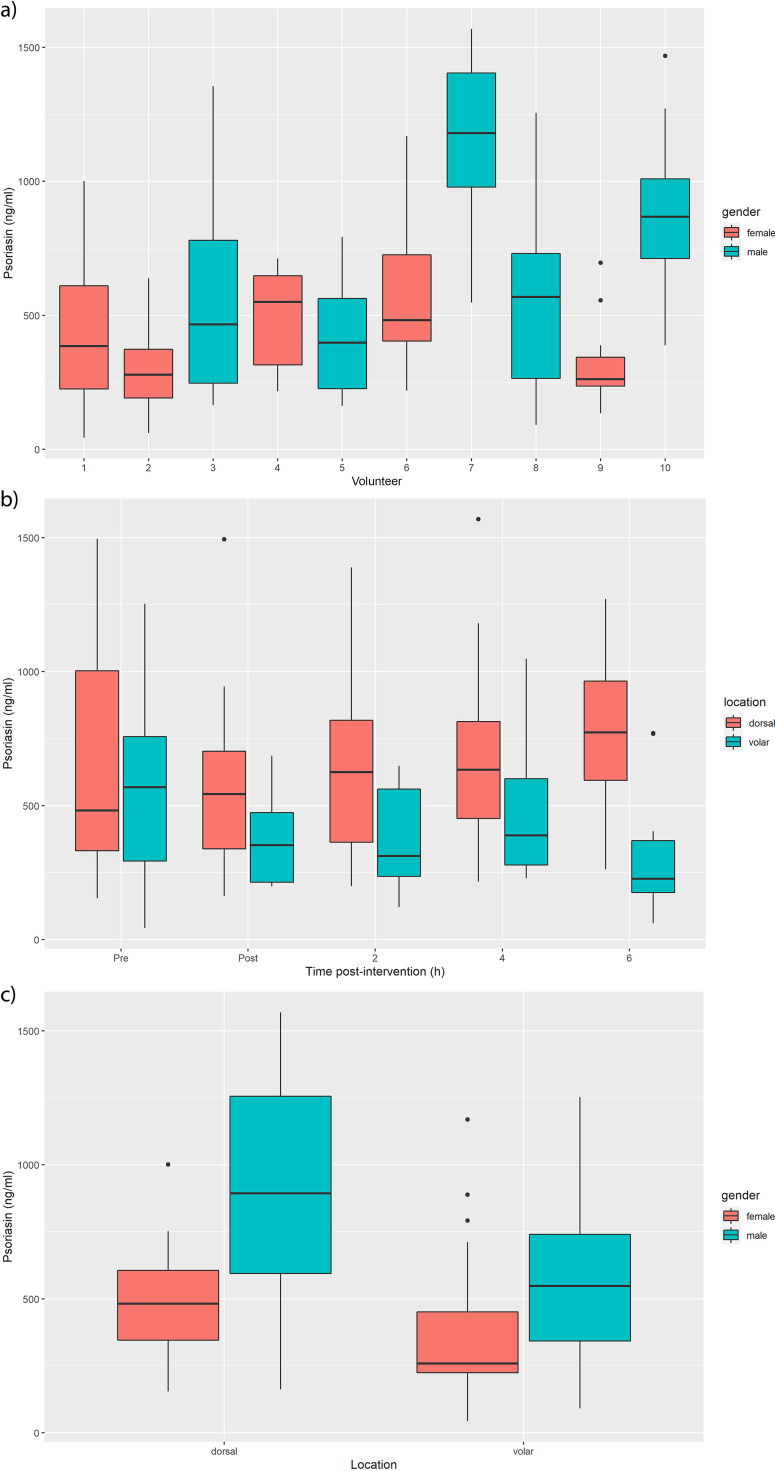
Psoriasin (a) per individual, (b) before and after wiping, and (c) per location for males and females. Psoriasin concentrations vary between individuals and both location and gender have apparent effects, although a larger study may be needed to confirm the significance of gender. The effect of the intervention is less clear, although a drop in levels can be observed in most individuals for the dorsal forearm.

Psoriasin concentrations quantified from 10 individuals by ELISA ranged from 42 to 1,569 ng/mL across the samples (Table S3). Concentrations were highly variable between individuals (Fig. 6a) and more pronounced than the effect of treatment (Fig. 6b). A drop was observed following ethanol wiping of the volar forearm (Fig. 6b), but recovery was not clear. The location had a significant effect (*P* = 0.037, *n* = 10, Wilcoxon paired test), with psoriasin concentrations higher for the dorsal forearm than the volar for most individuals (Fig. 6c). Males tended to have higher concentrations than females (Fig. 6a), although this was not as significant (*P* = 0.056, *n* = 20, Wilcoxon test) and was largely driven by individuals 7 and 10. Because of the variability between individuals, psoriasin concentrations were averaged across time, significantly reducing the power of the study. More data would be necessary to increase statistical significance, but overall, it seems that location and gender influenced psoriasin concentrations and individual variability outweighed the effect of treatment.

10.1128/msphere.00171-22.3 TABLE S3 Psoriasin concentration data in ng/mL for the (a) dorsal and (b) volar forearm showing that the psoriasin concentrations vary considerably between individuals, ranging from 42 to 1,569 ng/mL. Download Table S3, DOCX file, 0.02 MB. Copyright © 2022 Saville et al. 2022 Saville et al. https://creativecommons.org/licenses/by/4.0/ This content is distributed under the terms of the Creative Commons Attribution 4.0 International license.

## DISCUSSION

There is relatively little information available concerning potential positive or negative effects on the human host that can be causally linked to perturbing and/or remodeling of the skin microbiota by hygienic regimens and other anthropogenic processes. While changes in skin microbiota composition have been observed in some skin conditions, differentiating between cause and association remains a considerable challenge (17). The microbiota of the skin is regularly exposed to personal care products for cosmetic or hygienic purposes, during which an alteration in the composition and/or activities may occur either as the intended outcome or an unintended effect (17). Such processes could be mediated through direct interaction between the personal care product and the microbiota, or indirectly via interaction between the product and the epidermis. The epidermis forms an important antimicrobial barrier in healthy skin due to the physical structure and the production of antimicrobial peptides (27).

Establishing the timescales for skin microbiome reestablishment following perturbation, including effects on host factors, has been identified as a key step in consumer safety risk assessment for personal care product use (17). To generate data that can be generally applied in diligence-based risk assessment of personal care product use, we conducted a longitudinal study in which the microbiota of the dorsal and volar forearm skin was profiled before and after wiping with 70% ethanol. Ethanol was selected as a residue-free antimicrobial agent due to its rapid and profound effects on the viability of the skin microbiota and its being considerably less selective in action than other commonly used topical antimicrobials (28, 29). As a marker of the potential indirect effects of altering concentrations of host defense peptides, psoriasin, an antimicrobial peptide of host origin (4, 5, 22, 30), was measured throughout the study.

The epidermal microbiota was highly stable at baseline in individuals over three independent visits, with volar and dorsal sample profiles highly similar within individuals. These profiles are similar to those reported in an earlier study of the forearm microbiota (31) which showed it to be dominated by *Cutibacterium* and *Corynebacterium.* The presence of hair at the dorsal forearm sampling sites could have impacted swab collection, ethanol wiping, and the recolonization of the site. It has been proposed that the presence of appendageal structures, including hair follicles (32, 33) can contribute to the perpetuation of the skin microbiota (34). In agreement with investigations of antisepsis (35, 36), total eubacteria decreased significantly on exposure to ethanol but recovered in 6 h. Similar effects were observed for S. epidermidis, reaching minimum abundance in 2 h but with recovery taking longer (ca. 24 h). Microbial community profiles remained relatively stable despite topical ethanol application, although some transient compositional changes were apparent in a small number of individuals.

Relatively few investigations have applied deep sequencing to address the question of skin microbiome recovery after ethanol application. One key paper on this subject reported that topical treatment with antiseptics caused relatively in staphylococcal numbers were reported, which were associated with increased susceptibility to colonization by S. aureus (37). A subsequent investigation in humans reported considerable interpersonal variation in treatment effects on skin microbiome composition, and that organisms present at low abundance were more likely to be removed and replaced by organisms which were relatively abundant taxa before treatment (18). Concerning the host defense peptide psoriasin, no significant correlation could be found between microbiome diversity and psoriasin levels in this study; a larger cohort would be necessary to account for person variability. Data generally indicate a decrease in psoriasin concentrations upon swabbing, which rapidly recovered.

The fact that perturbation of the skin microbiota through UV radiation, friction, and washing would be characteristic of such microbial communities should not be overlooked. The hypothesis that once established, the skin microbiota is maintained by continuous endogenous inoculation has been previously evidenced. For example, (46) (2008), using swabs and biopsies to sample the skin microbiota, observed that swabbing was broadly comparable to more invasive methods in terms of bacterial diversity recovery. As proposed by Kong et al. (38), one attractive explanation for this is that the migration of differentiating skin cells, together with excretions from sweat glands, could transport bacterial cells continuously onto the skin surface, suggesting an important role for appendageal structures in this process. Costello et al. (34) reported on the disinfection of plots on the volar forearms and forehead, which were inoculated with tongue and skin microbiotas abstracted from other individuals. Between 2 and 8 h after this inoculation, the microbiome of forearms inoculated with microbiota samples from the tongue was more similar to tongue microbiomes than to the normal forearm microbiome. This was not the case for forehead plots that had been similarly inoculated with tongue material. The forehead was, therefore, more resistant than the forearm to microbiome perturbation of this type. It was hypothesized that sebaceous secretions were responsible for resistance to microbiota perturbation in the forehead sites. Therefore, the mechanisms underlying microbiome stability may include continuous re-inoculation from appendageal structures, inoculation from adjacent skin via squames and direct contact, and the presence of host-derived antimicrobial factors.

In summary, wiping the forearm with ethanol solution resulted in reduced bacterial abundance that was maximal after 2 h and took 6 h for recovery; and which, for S. epidermis, could take up to 24 h to recover, depending on the body site. Profiling of the forearm microbiome by sequencing indicated that the microbial composition was highly stable and, as observed by quantitative PCR (qPCR), it took approximately 6 h for the skin microbiome to normalize after treatment although, in the case of sequencing, this was significant only in females. Males generally had higher psoriasin concentrations than females and both males and females had a high amount of intrapersonal variation.

We assessed the skin microbiome effects of a single application of a broad-spectrum antimicrobial performed on three separate visits. In normal circumstances, however, the skin microbiome may be challenged more frequently due to daily, routine hand hygiene with soap or the application of alcohol-based disinfectants.

In conclusion, ethanol exposure elicited short-term, person-specific shifts in the volar and dorsal microbiomes and reductions in the absolute abundance of resident skin bacteria; some reductions in psoriasin concentrations were also observed. This work extends observations of skin microbiome stability and demonstrates resilience in a key antimicrobial peptide.

## MATERIALS AND METHODS

### Ethical approval.

This study has been approved by the research ethics committee at the University of Manchester (2017-0427-1760). All volunteers provided informed written consent and remained anonymous.

### Sample collection.

Samples were collected from the volar and dorsal forearms of 10 healthy volunteers (5 male and 5 female). All volunteers reported being healthy, without skin complaints, and aged 22 to 34. Volunteers were advised not to wash the sample area 8 h before baseline sampling and for the duration of the sampling appointment. Samples were collected using Catch-All swabs (Cambio, Cambridge, United Kingdom) and a custom-made polycarbonate template placed onto the sample area; this allowed collection of four adjacent samples from both the volar and dorsal forearm. There were four adjacent sample sites to allow samples to be taken at each time point without being influenced by prior swabbing. The template was set up so the sampling sites were longitudinal to the arm and adjacent to each other. This allowed each sample to include the area closest to both the wrist and cubital fossa/elbow and was deemed less variable than, for example, moving up the arm for each sample. Sterile phosphate-buffered saline was pipetted into the template and the area swabbed up and down 100 times. Swab heads were then placed into DNA extraction tubes from the PowerSoil kit (Qiagen, Manchester, United Kingdom).

Baseline samples were taken from the volar and dorsal forearm (termed “pre-wash”) using the first section in the template. The whole area was then cleaned using four 70% ethanol-soaked cotton wool pads (two for the volar and two for the dorsal forearm). The cleaning was performed as follows. The first ethanol-soaked cotton pad was used to wipe the area 20 times, in one direction from the cubital fossa/elbow toward the wrist, to reduce cross-contamination. The second ethanol-soaked pad was then used in an identical manner 5 times and the skin left to dry. The template was replaced in an identical position, and a sample was taken from the next adjacent section on the sample template (termed “post-wash”). Volunteers returned at two further time points during the sampling appointment and for three sampling appointments 1 week apart. The ethanol wash step described above was repeated at the beginning of each of the three appointments only, making a total of three sets of ethanol washes per area (volar/dorsal). The three appointments were to allow for the collection of samples at increasing time points post-alcohol wash. The same areas were sampled on each appointment; however, it was assumed that the week between appointments left enough time for the effects of the previous appointment to be minimized. The time points covered were as follows: Appointment 1: pre-wash, post-wash, 2 h, 4 h; Appointment 2: Pre-wash, post-wash, 4 h, 6 h; Appointment 3: pre-wash, post-wash, 6 h, 24 h. The whole procedure was carried out on both the volar and dorsal forearm.

### DNA extraction.

Bacterial genomic DNA was extracted using the PowerSoil kit (Qiagen) following the manufacturer’s instructions, with the addition of a 10-minute pre-incubation at 70°C followed by a 45-s beat-beating step using a FastPrep FP120 BIO101 (Thermo Fisher Scientific, Waltham, MA). DNA was extracted immediately from the swabs and then checked on a NanoPhotometer. Extracted DNA was amplified on the same day for the amplicon-sequencing PCR, and stored at −80°C before qPCR.

### Amplcon sequence analyses.

First-stage PCR was carried out using the 27F/338R primer pair, targeting the V1 to V2 region of the 16S rRNA gene. For the first-stage PCR, primers contained overhang (Illumina adapter) sequences (in **bold**), (27F: 5′-**ACACTCTTTCCCTACACGACGCTCTTCCGATCTNNNNN**AGAGTTTGATYMTGGCTCAG, 338R: 5′-**GTGACTGGAGTTCAGACGTGTGCTCTTCCGATCTT**GCTGCCTCCCGTAGGAGT). PCRs were carried out using the Hotstart Plus Mastermix kit (Qiagen, Manchester, United Kingdom) 6 μL extracted DNA, 2.5 μm forward primer, 2.5 μm reverse primer, 2.5 μL coral load, and 12.5 μL HotstarTaq (Qiagen). Samples (including positive and negative controls) were amplified in triplicate to reduce PCR bias, then pooled and purified with a PCR purification kit (Qiagen). The quality of DNA was checked using gel electrophoresis and samples were stored at −80°C. Amplification of the 16S rRNA gene was performed using the primer pair BAKT_341_F/BAKT_805_R with additional Illumina adaptor overhang nucleotide sequences. PCRs were performed using MyTaq Red Mix (Bioline, United Kingdom) and comprised 30 cycles, as follows: 5 min at 95°C, 30 sec at 95°C, 30 sec at 62.3°C, 90 sec at 72°C, and 7 min at 72°C. Samples were amplified in triplicate to minimize PCR bias and combined during further purification using a PCR purification kit (Qiagen, United Kingdom). Samples were sent (whole extract) on dry ice to the University of Liverpool Centre for Genomic Research to be sequenced on the Illumina MiSeq platform. (Paired-end 2 × 250 bp sequencing).

### Sequence data processing.

Raw sequencing reads (*n* = 15,180,915) from 240 samples were processed simultaneously as follows. PCR primers used for initial 16S rRNA gene amplification were removed from each fragment using Cutadapt (http://journal.embnet.org/index.php/embnetjournal/article/view/200) version 1.14 due to the presence of degenerate bases which may have impacted downstream taxonomic assessment. Sickle version 1.33 (https://github.com/najoshi/sickle) was used to quality trim DNA reads, using a minimum quality value of 28. Reads of less than 100 bp following quality trimming were discarded. If a single read was discarded during this process, its read pair was also discarded. Reads which passed filtering were merged using Pandaseq version 2.9 (39) to generate overlapping contigs with a minimum overlap of 20 bp and a minimum amplicon length of 200 bp. The resulting overlapped reads were de-replicated using Vsearch version 1.9.6 Linux x86-64 (https://github.com/torognes/vsearch) and searched against a BLAST database composed of the HOMD, HOMD extended, and previously described Greenegenes sequences (HOMDEXTGG) (40). Taxonomic classification was then performed as previously described (41) at 99% identity across 98% of the read length. Reads which were not classified by this process were discarded. This process resulted in 1,098 taxonomically classified OTUs. The resulting classification table and associated representative sequences, selected as the most abundant sequence for each classified taxa, were used as inputs for QIIME (Quantitate Insights into Microbial Ecology) version 1.9.1 (42). To perform the functional predictions, the OTUs were assigned taxonomies using the “assign taxonomy” script in QIIME, with the default settings and using SILVA release 123 as the reference database. Reads per sample varied between 30,322 and 88,709, with an average of 54,188 for 239 samples and 1 sample producing only 85 reads. Misclassified OTUs attributed to Plantae and Cyanobacteria were removed, as well as OTUs with low prevalence (i.e., those appearing in less than 10% of samples).

### Data analysis.

All the data were analyzed in an R environment. Because of inter-individual variability and to account for visits, unless stated otherwise, all the statistical tests used a hierarchical model with individuals and visits as random variables and time and location (dorsal/volar) as fixed variables (26). The richness analyses of the microbiome were carried out with Phyloseq (43) after rarefaction to a depth of 14,864 across all samples. To examine the effects of different factors, PCAs were plotted after centered log-ratio transformation and zero replacements of the non-rarefied tables to account for the compositionality of the data (44).

### Quantitative PCR.

To generate data on the absolute abundance of the skin microbiota, total populations of eubacteria were quantified, along with S. epidermidis, which was selected as a numerically important representative of the skin microbiota (3, 45). Quantitative PCR was carried out on the extracted DNA using the TaqMan probe BA04930791 (Life Technologies, United Kingdom) for total bacteria and compared to a standard curve generated from known quantities of DNA extracted from an S. epidermidis culture. A second probe specific to S. epidermidis BA04646141 (Life Technologies) was also used. qPCR cycling conditions for steps 3 to 4 were as follows: 40 cycles for 2 min at 50°C, 10 min at 95°C, 15 sec at 95°C, and 1 min at 60°C.

### Data availability.

The raw sequences have been deposited in the European Nucleotide Archive under accession no. PRJEB43062.
