# Supplementary material for: Transitory Shifts in Skin Microbiota Composition and Reductions in Bacterial Load and Psoriasin following Ethanol Perturbation
Source: mSphere. 2022 Jun 21;7(4):e00171-22. doi: 10.1128/msphere.00171-22 (PMC9429936; doi:10.1128/msphere.00171-22)
Supplement: TABLE S2 [file msphere.00171-22-s0002.docx]

**Table S2. Alpha-diversity for femalesFDR values obtained with MaasLin2 hierarchical models.** The location (dorsal and volar) and the timepoint (pre, post, 2h, 4h, 6h, 24h) were considered as fixed effects while the visit and the individual were considered as random effects.

| Gender | Timepoint | | | | | | | Location |
| --- | --- | --- | --- | --- | --- | --- | --- | --- |
| Sampling times | Pre-post | Pre-2h | Pre-4h | | Pre-6h | | Pre-24h | Dorsal-volar |
| Observed α-diversity | 0.109 | 0.109 | 0.021 | | 0.989 | | 0.989 | 0.109 |
| Shannon α-diversity | 0.109 | 0.122 | 0.021 | 0.989 | | 0.989 | | 0.109 |

Conclusions: observed alpha diversity decreases significantly (FDR at cut-off limit of 10%) with ethanol wiping and is recovered in 6h. The same trend is observed in Figure 3 for males but was not significant. The Shannon diversity exhibits a similar trend. The location has also a significant effect.
