# Supplementary material for: Transitory Shifts in Skin Microbiota Composition and Reductions in Bacterial Load and Psoriasin following Ethanol Perturbation
Source: mSphere. 2022 Jun 21;7(4):e00171-22. doi: 10.1128/msphere.00171-22 (PMC9429936; doi:10.1128/msphere.00171-22)
Supplement: TABLE S1 [file msphere.00171-22-s0001.docx]

**Table S1. qPCR t-tests, FRD** **values obtained with MaasLin2 hierarchical models. The timepoints (pre, post, 2h, 4h, 6h, 24h) were considered as fixed effects while the visit and the individual were considered as random effects.**

| Sampling times | Pre-post | Pre-2h | Pre-4h | Pre-6h |
| --- | --- | --- | --- | --- |
| Total eubacteria, volar | 0.077 | 0.077 | 0.077 | 0.544 |
| Total eubacteria, dorsal | 0.191 | 0.350 | 0.350 | 0.660 |
| *S. epidermidis,* volar | 0.858 | 0.858 | 0.858 | )0.858 |
| *S. epidermidis,* dorsal | 0.786 | 0.786 | 0.786 | 0.786 |

*Removed visit 3 of patient 7 as unlikely amplification.

Conclusions: numbers of both total eubacteria and *S. epidermidis* tend to drop with treatment but only significantly for the total volar. It recovers in 6 h.
