# Supplementary material for: Transitory Shifts in Skin Microbiota Composition and Reductions in Bacterial Load and Psoriasin following Ethanol Perturbation
Source: mSphere. 2022 Jun 21;7(4):e00171-22. doi: 10.1128/msphere.00171-22 (PMC9429936; doi:10.1128/msphere.00171-22)
Supplement: TABLE S3 [file msphere.00171-22-s0003.docx]

**Table S3. Psoriasin concentrations in ng/ml a) dorsal, b) vola forearm**

| a) | Individual | | | | | | | | | |
| --- | --- | --- | --- | --- | --- | --- | --- | --- | --- | --- |
| Time | 1 | 2 | 3 | 4 | 5 | 6 | 7 | 8 | 9 | 10 |
| Pre | 231 | 232 | 758 | 309 | 513 | 1169 | 1177 | 570 | 329 | 529 |
| Post | 225 | 204 | 353 | 238 | 686 | 399 | 548 | 198 | 199 | 388 |
| 2hr | 166 | 121 | 236 | 359 | 563 | 559 | 611 | 264 | 235 | 649 |
| 4hr | 379 | 639 | 229 | 321 | 398 | 888 | 1048 | 264 | 243 | 486 |
| 6 hr | 225 | 61 | 165 | 262 | 229 | 405 | 769 | 209 | 134 | 770 |
| 24hr | 98 | 71 | 508 | 550 | 333 | 451 | 717 | 91 | 136 | 733 |
| Pre 2 | 43 | 98 | 748 | 712 | 502 | 699 | 1208 | 462 | 258 | 820 |
| Pre 3 | 252 | 293 | 684 | 724 |  | 792 | 1095 | 568 | 246 | 1253 |
| b) | Individual | | | | | | | | | |
| Time | 1 | 2 | 3 | 4 | 5 | 6 | 7 | 8 | 9 | 10 |
| Pre | 1001 | 154 | 181 | 682 | 223 | 403 | 1447 | 594 | 388 | 894 |
| Post | 630 | 359 | 332 | 613 | 162 | 472 | 1494 | 727 | 265 | 945 |
| 2hr | 644 | 417 | 846 | 606 | 199 | 345 | 1389 | 735 | 300 | 1005 |
| 4hr | 727 | 536 | 424 | 216 | 226 | 571 | 1569 | 1181 | 696 | 843 |
| 6 hr | 490 | 263 | 873 | 708 | 793 | 752 | 1184 | 996 | 556 | 1272 |
| 24hr | 603 | 319 | 1356 | 555 | 785 | 219 | 1361 | 531 | 378 | 1009 |
| Pre 2 | 391 | 346 | 250 | 482 |  | 482 | 1143 | 1256 | 288 | 1468 |
| Pre 3 |  | 461 | 861 |  |  |  | 1496 |  | 237 | 1566 |

Conclusions: Psorisain levels vary considerably between individuals ranging from 42 to 1569 ng/ml.
